# Synthesis and Characterization of Iodinated Chitosan Nanoparticles and Their Effects on Cancer Cells

**DOI:** 10.1155/2024/3850286

**Published:** 2024-09-02

**Authors:** Germán Alvarado Tenorio, Roberto Espinosa Neira, Carlos Alberto Ávila Orta, Gabriela Yolotzín Romero Zúñiga, Hortensia Ortega Ortiz

**Affiliations:** Departamento de Materiales Avanzados, Centro de Investigación en Química Aplicada Blvd, Enrique Reyna Hermosillo No. 140 CP, Saltillo 25294, Coahuila, Mexico

## Abstract

The high degree of chemical modification of the chitosan chains due to protonated amine groups allows them to react with many negatively charged surfaces as anionic polymers and cell membranes, resulting in an attractive material for medical and pharmaceutics applications. Incorporating ionic iodine (I^−^ and IO_3_^−^) on chitosan chains is a direct way to successfully obtain chitosan-iodine nanoparticles (CSNPs-I and CSNPs-IO_3_) through ionic gelation. The nanoparticles (NPs) present a hemispherical morphology with sizes around 30–70 nm for CSNPs-I and CSNPs-IO_3_, similar to chitosan NPs, in accordance with SEM and DLS techniques. The XRD characterization did not show noticeable differences in the crystallinity index (CI) for CSNPs and CSNPs-I, 48.4 and 49.3%, respectively, but for CSNPs-IO_3_, the CI decreased to 43.85%. The cytotoxic effects on human tumor cells of chitosan and iodine-modified chitosan nanoparticles (CSNPs-I and CSNPs-IO_3_) were evaluated for 24 h in a range from 0.15 mg/mL to 0.95 mg/mL concentrations, where CSNPs-IO_3_ presented the lower viability for lung cancer A549, followed by cervical cancer HeLa cell and finally breast cancer MDA-MB-231, with a weight content of iodate ion in a range of 8.7 to 15 *μ*g. This work presents the possibility of exploring chitosan-iodine NPs in medical applications.

## 1. Introduction

Chitosan (CS) is a natural linear polycationic copolymer of 2-amino-2-deoxy-*β*–D-glucopyranose, obtained by alkaline deacetylation of chitin. Chitin is present in crustaceans, mollusks, insects, and fungi; hence, it is the second most abundant natural polysaccharide [1]. The protonation of amine groups (RNH_3_^+^) in the acid media of CS results in a soluble and highly positively charged, along with the –OH groups, enabling a high degree of chemical modification of the chains [2]. The amino groups can react with many negatively charged surfaces, such as anionic polymers and cell membranes [3].

Chitosan is often compared with chitooligosaccharides (COS), which are oligosaccharides derived from the depolymerization of chitin or chitosan. COS consist of short chains of glucosamine monomers linked by glycosidic bonds and exhibit various beneficial biological properties such as antimicrobial, antioxidant, and anti-inflammatory activities. However, compared to COS, CS typically exhibits higher viscosity, leading to the development of more stable gels. Moreover, due to its larger molecular size, chitosan has enhanced capacity for encapsulating bioactive molecules such as drugs and nutrients, protecting them from degradation and enabling controlled release.

The excellent properties of chitosan, such as nontoxicity, biocompatibility, biodegradability, and low cost, are of commercial importance in medical and pharmaceutics applications, such as drug release in cancer disease [4–8]. There are many reports about the antitumoral activity of chitosan, the mechanism of which is related to membrane-disrupting and apoptosis-inducing activities [9–14]. It is a cationic polysaccharide, which means it has a positive charge, making it attractive for forming complexes with negatively charged drugs. The positive charge of CH allows it to bind to negatively charged molecules, such as nucleic acids, proteins, and drugs. This property also makes it an attractive vehicle for gene therapy and for delivering drugs to target cells or tissues. Additionally, CH has several advantages as a drug delivery system, including its biocompatibility, nontoxicity, biodegradability, its ability to protect drugs from degradation, and its ability to target specific cells or tissues [15].

Several modifications of chitosan have been demonstrated to enhance solubility and biological activity, including derivations due to amino groups and acetamido residues [16]. Additionally, high and low molecular weight chitosan has demonstrated cytotoxicity in breast cancer MCF-7, cervical cancer HeLa, and osteosarcoma SaOS-2 cell lines but less cytotoxicity on dermal fibroblasts [10, 17]. It has been reported that increased concentrations of chitosan inhibit the migration of breast cancer cells. MDA-MB-231 through a Matrigel-coated membrane is inhibited by increased concentrations of chitosan [18]. Additionally, chitosan nanoparticles (CSNPs) have been demonstrated to suppress the proliferation of human hepatoma BEL7402 cells through cell necrosis via neutralization of its surface charge, cell membrane penetration, MMP reduction, and lipid peroxidation induction *in-vitro* [19]. Besides, molecular iodine has presented a suppressive effect related to breast cancer cells, which may occur in two ways: directly dissipate the potential of the mitochondrial membrane (mitochondrial apoptosis) and indirectly through iodo-lipid formation and the activation of gamma-type peroxisome proliferator-activated receptors, causing apoptosis or differentiation [20, 21]. The breast shares with the stomach and thyroid, an important iodide-concentration and peroxidase activity, where diseases have been reported caused by dietary iodine deficiency [22–24]. On the other hand, Bigoni-Ordóñez et al. [25] reported that I_2_ on monolayers or cervospheres of HeLa and SiHa cell lines derived from cervical cancer was able to inhibit tumors in mice.

On the other hand, Hailfed et al. [26] recently reported the use of NPs containing iodine (Niodx). It is a 20-nm nanoparticle with a long blood half-life of 40 h and is well tolerated at an intravenous dose of 7 g iodine/kg (in mice). It accumulates in gliomas and brain metastases and provides robust tumor imaging, potentially useful for diagnostic purposes and alignment for surgeries or radiotherapy. High tumor loading with iodine results in absorbing more X-rays during radiotherapy, thus boosting the local dose at the tumor several-fold, which is shown to provide significant life extension compared to radiotherapy alone. The same working group also reported this methodology with breast cancer cells [27].

Until now, only the effects of CSNPs and iodine on some cancer cells have been reported separately; in addition, studies on the effects of chitosan nanoparticles (CSNPs) and iodine have primarily focused on individual cancer cells. This study represents a significant advancement by synthesizing iodinated chitosan nanoparticles via the gelation method, followed by comprehensive characterization and evaluation of cytotoxicity in three tumor cell lines: MDA-MB-231 breast cancer, HeLa cervical cancer, and A549 lung cancer. This approach not only enhances understanding of the combined effects of chitosan and iodine across different cancer types but also provides a critical perspective for their potential medical application, contributing to the development of innovative and more effective cancer therapies.

## 2. Experimental Section

### 2.1. Materials and Methods

Chitosan of viscosity molecular weight 200,000 g/mol and degree deacetylation of 98% was purchased from Marine Hydrocolloids, Kerala, India. Sodium tripolyphosphate anhydrous (TPP) was acquired from Sigma Aldrich. Iodine salts, potassium iodide (KI), potassium iodate (KIO_3_), and acetic acid glacial ACS were purchased from Fermont. Thermo SCIENTIFIC SORVALL ST 16R (8500 rpm) and Allegra 64R (15000 rpm) centrifuges were used for the centrifugation. A BRANSON Digital Sonifier 250, model 102C (CE), was used at 70% amplitude. The samples were freeze-dried by LABCONCO FreeZone 1 Model 7740021. To determine iodine content in NPs, 0.5 g of dry sample was digested in 2 M KOH before ICP (Inductively Coupled Plasma) analysis.

### 2.2. Cell Cultures

The human breast cancer cells MDA-MB-231, cervical cancer cells HeLa, and lung cancer cells were provided by ATCC and cultured in the DMEM-F12 (Corning, Manassas, USA) medium supplemented with 10% fetal bovine serum (FBS, Corning, USA) and antibiotic-antimycotic solution (Sigma-Aldrich, USA) in a humidified atmosphere containing 5% CO_2_ and 95% air at 37°C. Cell cultures were seeded 24 h before treatment.

### 2.3. Nanoparticle Synthesis

The 0.5% (w/v) chitosan solution was prepared by dissolving chitosan powder in 1% (v/v) acetic acid until the solution was transparent. Subsequently, the solution was filtered and refrigerated before being used in the reactions. After that, sodium tripolyphosphate (TPP) was dissolved in deionized water at a concentration of 0.5% (w/v). A 0.1 M solution of KI and KIO_3_ was prepared to synthesize the iodine complexes CSNPs-I and CSNPs-IO_3_, respectively.

For preparing chitosan and chitosan-iodine NPs, the ionic gelation method was used with TPP as a cross-linking agent in a 10 : 3 (v/v) ratio of chitosan with respect to TPP [28]. For the formation of CSNPs, TPP was slowly dripped and the reaction was kept under stirring for 2 h at 500 rpm. Subsequently, the reaction was precipitated by centrifuging at 8,500 rpm for 30 min; the supernatant was removed and centrifuged at 15,000 rpm for 30 min to precipitate the NPs formed. Subsequently, the NPs were dispersed in deionized water to remove excess unreacted material; washing was performed twice with centrifugation at 15,000 rpm for 30 min. Finally, the precipitated material was freeze-dried. For the formation of CSNPs with iodine, after 2 h of reaction of the CSNPs had elapsed, 0.24 mL of the KI or KIO_3_ solution was added dropwise for each milliliter of chitosan solution. The reaction was left stirring for 24 h to finally apply the same centrifugation, washing, and drying methodology as the CSNPs.

The synthesized CSNPs with iodine and without iodine were characterized by dynamic light scattering (DLS), FTIR-ATR, X-ray diffraction (XRD), thermogravimetric analysis (TGA), differential scanning calorimetry (DSC), and scanning electron microscopy (SEM).

Dispersions were prepared in deionized water to determine the size of the chitosan and chitosan-iodine NPs. 5 mg of each sample was weighed and stirred in 50 mL of deionized water. Subsequently, the samples were sonicated for three cycles of three min in ultrasound, and measurements were immediately made to avoid precipitation.

### 2.4. Cytotoxicity Assay

**Scheme 1:**
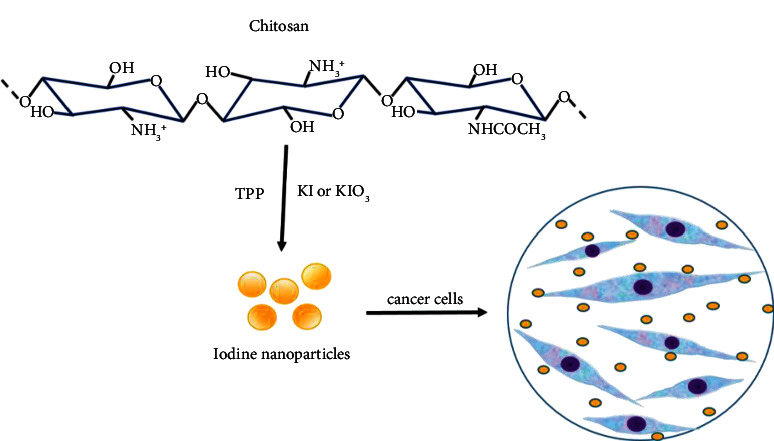
Cytotoxicity assay.

Culture cells were grown in 96-well plates (7500 cells/well) and treated with different doses of CSNPs. Cell viability was then evaluated using 3-(4, 5-dimethylthiazol-2-yl)-2, 5-diphenyltetrazolium bromide) (MTT) assay (7500 cells/well) [29], and 20 *µ*L of MTT 5 mg/mL was added to each well and incubated with MTT (Sigma-Aldrich, USA). The supernatant was carefully removed, 150 *µ*L of acid-alcohol was added, and the plates were shaken on an orbital shaker for 15 min. O.D. was read at 570 nm. The results are expressed as the mean ± SD. Data were statistically analyzed using one-way ANOVA or two-way ANOVA followed by Dunnett´s multiple comparison test; the statistical probability of *P* ≤ 0.05 was considered significant.  Scheme 1 describes the procedure to realize the cytotoxicity assay after synthesizing chitosan nanoparticles-iodine using gelation.

## 3. Characterization

### 3.1. Dynamic Light Scattering (DLS)

A dynamic light scattering MICROTAC (Nanotrac wave II Q) and Zeta-Check Particle Charge Reade were used to determine the mean size diameter of NPs. A dispersion with a concentration of 5 mg in 20 mL of deionized water was sonicated to make three measurements for each sample.

### 3.2. Infrared Spectroscopy by ATR (FTIR-ATR)

The spectrophotometer Nicolet model iS50 FTIR with Attenuated Total Reflectance (ATR) was employed in a range of 400–4000 cm^−1^ to determine the chemical composition of powder samples without prior drying.

### 3.3. X-Ray Diffraction (XRD)

The XRD spectra of NPs were obtained with diffractometer D8 Advanced ECO BRUKER, in the 2*θ* ranges of 5–50°, 0.02° step resolution, and 1 s per step, under 40 kV voltage, 25 mA current, Cu–K, and 1.54060 Å. The crystallinity index (CI) of NPs was calculated from the height ratio between the intensity of crystalline peak (I_200_) and total intensity, CI =  (I_200_ − I_AM_/I_200_) ∗ 100, after subtraction of the background without CSNPs. Debye Scherrer's equation, *D* = *kλ*/*β*Cos*θ*, was used to determine the crystallite size, where

*D* = crystallite size (nm).

*k* = Scherrer constant (0.9).

*λ* = Wavelength X-ray.

*β* = FWHM (radians).

*θ* = Peak (radians).

The *d*-spacing was calculated by Bragg's law, *d* = *n λ*/2sin*θ*, where

*d* = *d*-spacing.

*λ* = X-ray wavelength.

*n* = order of reflection.

*θ* = Bragg's angle (radians).

### 3.4. Thermogravimetric Analysis (TGA) and Differential Scanning Calorimetry (DSC)

The thermogravimetric analysis was performed by TA Instruments Q500, and the thermal transition was performed by using the differential scanning calorimeter using TA Instruments 2500, at a 10°C/min rate under nitrogen, for both techniques. The samples were heated from 25°C to 600°C for TGA and from 25°C to 300°C for DSC.

### 3.5. Scanning Electronic Microscopy (SEM)

For morphological characterization and size of CSNPs and chitosan-iodine NPs, Field Emission SEM JEOL 7800F was performed. The samples were dispersed in deionized water and sonicated for 5 min. Then, a few drops were placed on an aluminum pin and dried at room temperature to examine. An acceleration voltage of 2 kV with magnifications of 150,000x and WD = 3.5 mm was used.

### 3.6. Iodine Content Determination by ICP

The alkaline ash technique was used to determine iodine content [30]. The dry samples were predigested with 2 mL of 2 M KOH and 1 mL of 2 M KNO_3_ in a stove at 100°C for 2 h. Then, the samples were placed in the crucible in a muffle furnace at 580°C for 3 h. Then, the ashes were dissolved in 2 mL of 2 M KOH. The samples were centrifuged, and finally, 1 mL of the supernatant was taken to be adjusted to 10 mL with 2 M KOH for their quantification in an Agilent 725 ICP-OES.

## 4. Results and Discussion

### 4.1. Nanoparticle Size Determination

**Figure 1:**
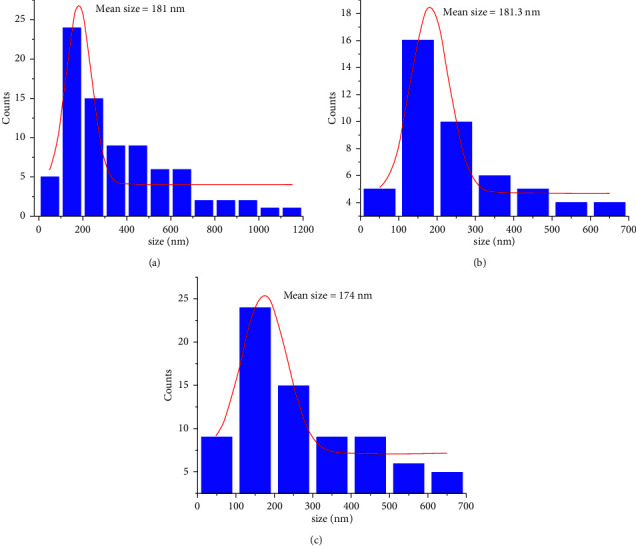
Gaussian normal distribution of CSNPs with iodine and without iodine, CSNPs (a), CSNPs-I (b), and CSNPs-IO_3_ (c).

Once the measurements in triplicate were obtained by MICROTAC DLS equipment, the average was plotted. Then, a Gaussian fit with Origin 9.0 Software was applied to the size distribution of NPs. It can be observed that NPs with and without iodine present an average size of 180 nm. However, a broader distribution is observed for CSNPs, with sizes slightly larger even up to 1.2 nm (Figure 1(a)), in comparison to chitosan-iodine NPs, where the incorporation of I^−^ and IO_3_^−^ ions on chains of chitosan allowed a decrease in size of agglomerates, due possibly to the repulsion between NPs, which favor the dispersion in water Figures 1(b) and 1(c). This was observed at the moment of making the dispersion by sonication, where less agglomerates and smaller were observed in the CSNPs-IO_3_ dispersion for DLS characterization.

### 4.2. Study of Chemical Composition by FTIR

**Figure 2:**
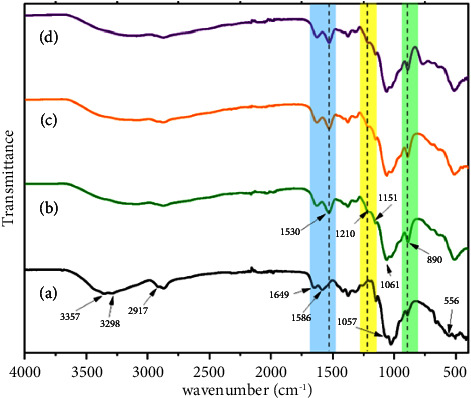
FTIR of chitosan (a) and nanoparticles, without iodine (b) and with iodine, CSNPs-I (c) and CSNPs-IO_3_ (d).

In Figure 2(b), the Nicolet FTIR spectra present peaks localized at 1210 cm^−1^, corresponding to ─P=O stretching vibration (yellow zone), 1151 cm^−1^ to symmetrical and asymmetrical stretching vibration of PO_2_ groups, at 1061 cm^−1^ symmetric and asymmetric stretching vibration of PO_3_ groups, and 890 cm^−1^ attributed to P-O-P asymmetric stretching (green zone). This confirms the presence of TPP in chitosan chains, indicating cross-linking and NPs formation, as reported [31] compared to raw chitosan in Figure 2(a). These peaks were also observed in samples after adding iodine in synthesis, Figures 2(c) and 2(d). This indicates that the formation of CSNPs remained even in the presence of iodine ions, which interact with CSNPs to form iodine nanoparticles' complex, either with iodide or iodate ions (peaks not observable in the spectrum). For chitosan, the band at 1586 cm^−1^ is attributed to ─N-H deformation of amine groups, which disappear in chitosan nanoparticles and chitosan-iodine NPs, and a new band appears at 1530 cm^−1^, refer to NH_3_^+^, indicating cross-linking for all NPs, due to interaction of phosphate groups negatively charged and amine groups (blue zone in Figure 2). The peaks localized at 3357 cm^−1^ (–OH and –NH_2_ stretching), 2917 cm^−1^ (-CH stretching), 1649 cm^−1^ (amide I), 1057 cm^−1^ (C-O-C stretching), and 556 cm^−1^ (pyranose ring stretching vibration) correspond to chitosan powder, Figure 2(a).

**Figure 3:**
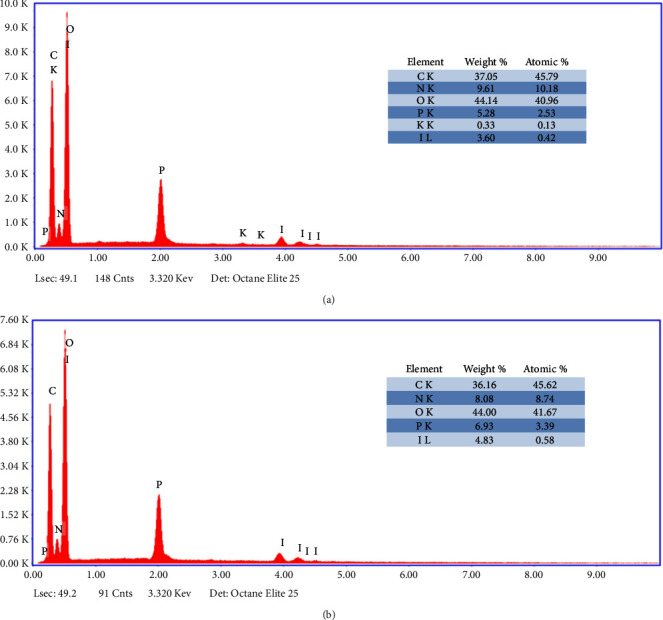
Análisis EDAX de las muestras CSNPs-I (a) and CSNPs-IO_3_ (b).

Due to iodine's transitions not occurring within the wavelength range covered by infrared spectroscopy (approximately 4000−400 cm⁻^1^), it is not possible to determine iodine using FTIR. Additionally, an EDAX study was conducted as a complementary analysis to analyze the chemical composition of the CSNPs. The results obtained are shown in Figure 3, and both samples were found to contain approximately 3 to 5% iodine.

### 4.3. Crystallinity of Nanoparticles

**Figure 4:**
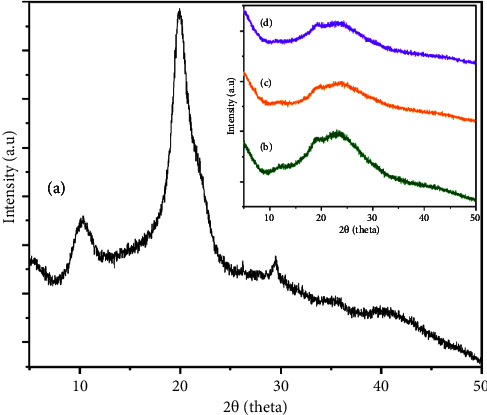
XRD patterns of chitosan (a), CSNPs (b), CSNPs-I (c), and CSNPs-IO_3_ (d).

Figure 4(a) shows the pattern of representative peaks of chitosan at 2*θ* = 10.3°, 20°, and 29.5°, indicating its semicrystalline structure [32, 33]. The NPs (Figure 4(b)) exhibited a reduction and broadening of the peaks, resulting in two broad peaks at 2*θ*: the first (much smaller) from 11° to 12.5° and the second from 22° to 24°. Additionally, a new peak at 19.2° can be observed. These differences are related to the incorporation of TPP groups and are widely reported for the synthesis of chitosan nanoparticles [34]. The peak positions for the CSNPs-I and CSNPs-IO_3_ samples did not show significant changes, indicating that the spacing of the chitosan NPs chains did not increase; however, this is evidence that all samples are amorphous materials. The most significant change in the iodine-containing samples is the intensity and width of the peaks. As observed, the intensity of the peak ranging from 20° to 25° in 2*θ* was notably reduced (wider) for both samples, CSNPs-I and CSNPs-IO_3_. This indicates the distribution of I⁻ and IO_3_^−^ ions along the chitosan nanoparticle chains and suggests that they are being “trapped” or “interaction” in the NPs. In fact, the peak at 2*θ* from 19° to 26° for the CSNPs-IO_3_ sample (Figure 4(d)) appears slightly less intense and wider than that of CSNPs-I, which may be related to the size of the IO_3_^−^ ion. The phenomenon of “trapping” or “encapsulation” of drugs in biomaterials, such as NPs of chitosan, alginate, among others, has been previously reported [35–37]. To support this assertion, the crystallinity index was calculated. The crystallinity index calculated for CSNPs and CS-I nanoparticles was similar, 49.3% and 48.4%, Figures 4(b) and 4(c), respectively. However, when the IO_3_^−^ ions were incorporated in CSNPs synthesis, the CI decreased to 43.85%, Figure 4(d). These values indicate an amorphous nature due to the cross-linking or “encapsulation” of CS with TPP and the interaction of iodate ions with CSNPs, compared to the 69.9% crystallinity of raw chitosan, Figure 4(a). The difference in crystallinity between CSNPs-I and CSNPs-IO_3_ could be due to the interaction of IO_3_^−^ ions with amino groups in the chain polymer, which avoid packing closely and then decrease the crystallinity compared to I^−^ ion interaction [37]. In Table 1, the size of the crystallite and d-spacing are shown.

### 4.4. Thermogravimetric and Differential Scanning Calorimetry Analysis

**Figure 5:**
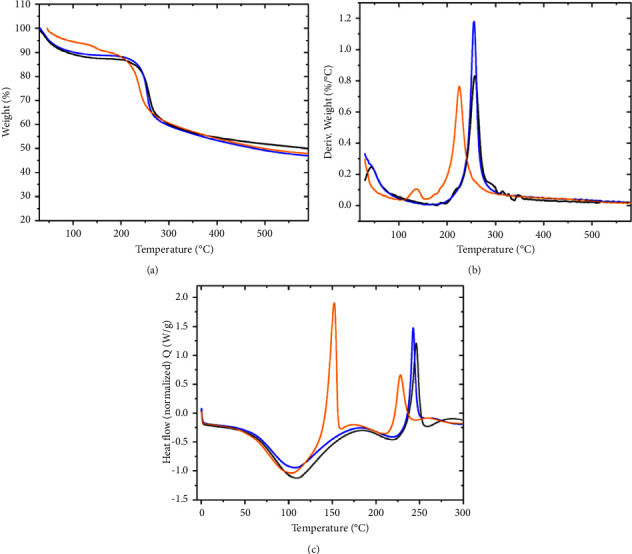
Thermogravimetric analysis (a, b) and differential scanning calorimetry (c) of CSNPs (black line), CSNPs-I (blue line), and CSNPs-IO_3_ (orange line) nanoparticles.

TGA plots in Figure 5(a) show that in all NPs, the weight decreases with increasing temperature, but from 25 to 210°C, the weight loss of the CSNPs-IO_3_ nanoparticles (orange line) is greater than that of the nanoparticles of CSNPs (black line) and CSNPs-I (blue line) and the highest percentage of weight loss occurs at a lower temperature (230°C); therefore, CSNPs-IO_3_ nanoparticles are less stable.

This may be due to the fact that the iodate groups are more voluminous and require less energy to break the electrostatic interaction they had with the protonated amino groups of the CSNPs.

The TGA curves for the CSNPs, CSNPs-I, and CSNPs-IO_3_ samples are shown in Figure 5(a). The thermograms indicate that the first stage of mass loss for all samples occurs in the range of 25 to 150°C, which is primarily attributed to volatile components and water evaporation. According to the data obtained from the DTG curves Figure 5(b), it is observed that the water content of the CSNPs-I sample has an intermediate value similar to that of CS and slightly higher for CSNPs with a value of 12.7%. For the CSNPs-IO_3_ sample, there is a stage in the temperature range of 25–100°C, with a mass loss of 6.2%, and another stage at a temperature of 147.8°C, with a mass loss of 3.4%. Summing the two mass losses from these stages results in a total of 9.6%, the lowest water content of all samples. This likely indicates the interaction of iodate ions with the amino groups that remained available after adding TTP in the formation of chitosan nanoparticles, leading to lower affinity for water molecules in the CSNPs-IO3 sample, as amino groups are known to have a hydrophilic character.

In the second stage (160–400°C), the degradation temperature (Td) obtained from the graphs in Figure 5(b) for the CSNPs and CSNPs-I samples shows similar intermediate values but lower than 288.7°C, which corresponds to chitosan. The CSNPs-IO_3_ sample exhibits the lowest thermal stability of all the samples, with a Td of 238.4°C. At this degradation temperature, dehydration of saccharide rings, depolymerization, and decomposition of acetylated and nonacetylated units of the polymer occur [38]. For CSNPs, in addition to the Td of 256.9°C, another Td at 347.5°C is present, which could correspond to chitosan cross-linked with TTP.

In the analysis of the DSC thermograms, the CSNPs and CSNPs-I samples exhibit three peaks: two endothermic and one exothermic. The first endothermic reaction corresponds to dehydration, as the polar groups make the structure more stable due to the interaction between the phosphate groups of TPP and the amino groups of CS. For the first endothermic peak of the CSNPs sample, water loss occurs at a temperature range of 57.8 to 191.5°C, while for the CSNPs-I sample, it ranges from 57.4 to 189°C. For the second endothermic peak, the CSNPs sample ranges from 192 to 220°C and the CSNPs-I sample ranges from 190 to 220°C. This second endothermic peak is related to the loss of hydrophilic groups. Degradation begins with the exothermic reaction at a temperature range of 220 to 280°C for the CSNPs sample and 220 to 260°C for the CSNPs-I sample.

The DSC thermogram of the CSNPs-IO_3_ sample shows one endothermic reaction and two exothermic reactions. Water loss occurs between 52 and 140°C. The first exothermic peak is located in the range of 143–177°C, while the second one is in the range of 228–258°C. The second exothermic peak is related to the introduction of phosphate groups from TPP into CS, while the first exothermic peak is believed to be related to the introduction of iodate ions into the chitosan nanoparticles.

### 4.5. Morphology of Nanoparticles

**Figure 6:**
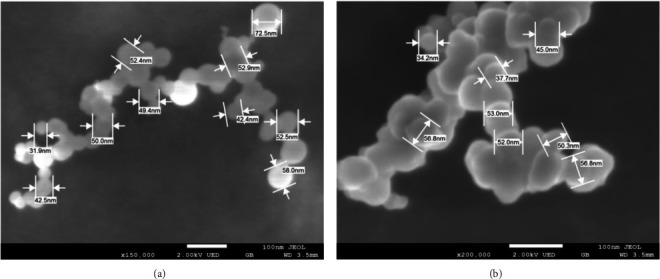
SEM of CSNPs with iodide, hemispherical particles (a), and irregular forms in (b).

SEM images confirm the formation of chitosan-iodine nanoparticles, which appear as small and individual hemispherical particles with a diameter of around 30–70 nm (Figure 6(a)). The agglomerates in Figure 6(b) indicate that the interaction between NPs occurred, as observed in DLS characterization, where the sizes have higher values. The micrographs in Figures 6(a) and 6(b) correspond to the CSNPs-I sample. Similar morphologies and sizes were present for the CSNPs-IO_3_ and CSNPs samples (micrographs are not shown).

### 4.6. Iodine Content Determination

The basic digestion was realized and quantified in a spectrophotometer Agilent 725 ICP-OES to corroborate the presence of iodine on CSNPs. For approximately 0.5 gram of sample, the content of I^−^ and IO_3_^−^ ions obtained were 16343.60 mg·kg^−1^ and 15828.20 mg·kg^−1^, respectively. Then, for each gram of the CSNPs-I complex, there are 16.47 milligrams of I^−^ ion, and for each Gram of the CSNPs-IO_3_ complex, there is 15.86 mg of IO_3_^−^. Therefore, for the concentrations used in the cytotoxicity assay (0.15, 0.35, 0.55, 0.75, and 0.95 mg·mL^−1^) of chitosan and chitosan-iodine nanoparticles and its corresponding content of I^−^ or IO_3_^−^ were calculated and shown in Table 2.

### 4.7. Cytotoxicity

**Figure 7:**
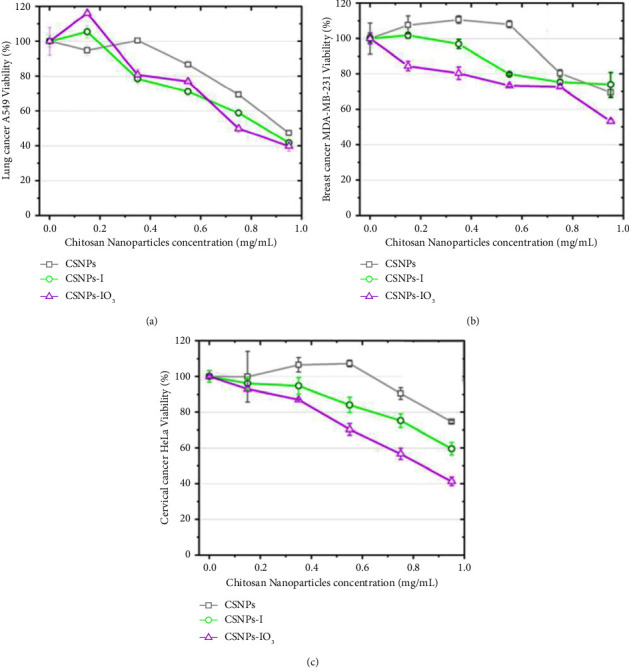
Effect of CSNPs viability in human tumoral cell lines: (a) lung cancer A549, (b) breast cancer MDA-MB-231, and (c) cervical cancer HeLa cell lines were evaluated during 24 h with an MTT assay. The graphs represent the mean ± SD and expressed in cell viability percent. Data were analyzed using one-way ANOVA followed by Dunnett's multiple comparison test.

The cytotoxic effect of all CSNPs synthesized (CSNPs, CSNPs-I, and CSNPs-IO_3_) in human tumor cell lines was evaluated. Each cell line was treated with doses of 0.15, 0.35, 0.55, 0.75, and 0.95 mg/mL for 24 h (Figure 7).

A dose-dependent cytotoxic effect was observed for all CSNPs in all tumor cells. For lung cancer, A549 treated with CSNPs, CSNPs-I, and CSNPs-IO_3_ (gray, green, and purple lines, respectively) showed their minimum cell viability between 40 and 50% (0.95 mg/mL) (Figure 7(a)). All concentrations of CSNPs-I and CSNPs-IO_3_ showed a similar line trend of cytotoxicity, but CSNPs treatment showed slightly less toxicity. Breast cancer cells treated with CSNPs-IO_3_ showed more toxicity at 0.95 mg/mL (52% of cell viability) than CSNPs-I or CSNPs, 74 and 70%, respectively (Figure 7(b)). Finally, cervical cell cultures (HeLa) treated with CSNPs-IO_3_ showed 40% cell viability, cells treated with CSNPs-I showed 60% cell viability, and cells treated with CSNPs showed 75% cell viability Figure 7(c).

The difference in cytotoxicity for CSNPs-IO_3_ samples is more noticeable in HeLa, very possibly due to the presence of atoms of oxygen in the IO_3_^−^ ion, which could present catalytic activity inside living cells or promote the release of reactive oxygen species (ROS). Limbach et al. [39] reported the study of intracellular oxidations using iron, cobalt, manganese, and titanium on silica NPs as matrix and its corresponding pure oxides. The results showed that the NPs induced higher oxidative stress on lung epithelial cells (A549) than counterpart salts. In other work, ZnO nanoparticles presented a cytotoxic effect on the human glioma cell lines A172, U87, LNZ308, LN18, and LN229, breast (MCF-7) and prostate cancer (PC-3), caused by an enhancement in the generation of ROS in glioma cells, contributing to the apoptotic effect [39]. ROS includes the superoxide radical (O_2_^−^), hydrogen peroxide (H_2_O_2_), and the hydroxyl radical (^**·**^OH), which cause damage and cell death. Chitosan-assembled zinc oxide nanoparticle was reported as anticancer against cervical cancer cells, where the authors reported that the cytotoxicity was mainly attributed to ROS [40]. In our case, the positive surface of CSNPs and chitosan-iodine nanoparticles (zeta potential not shown) allowed its interaction with the anionic nature of cancer cells, and for the case where there is the presence of IO_3_^−^ ion, a major cytotoxic effect was exerted on HeLa, suggesting intracellular oxidation in this case. However, when CSNPs-I was used, the cytotoxic effect decreased but higher than that of CSNPs, indicating the contribution of iodine on cytotoxicity, as has been presented in various reports associated with the use of the iodine on different types of cancer, principally breast cancer [38–44]. Therefore, according to cytotoxicity results, intracellular oxidation could occur due to oxygen and/or suppressive effects caused by iodine. To corroborate the above-mentioned, it is necessary to realize assays to clarify the mechanism of cell death, which is out of the scope of this study.

**Figure 8:**
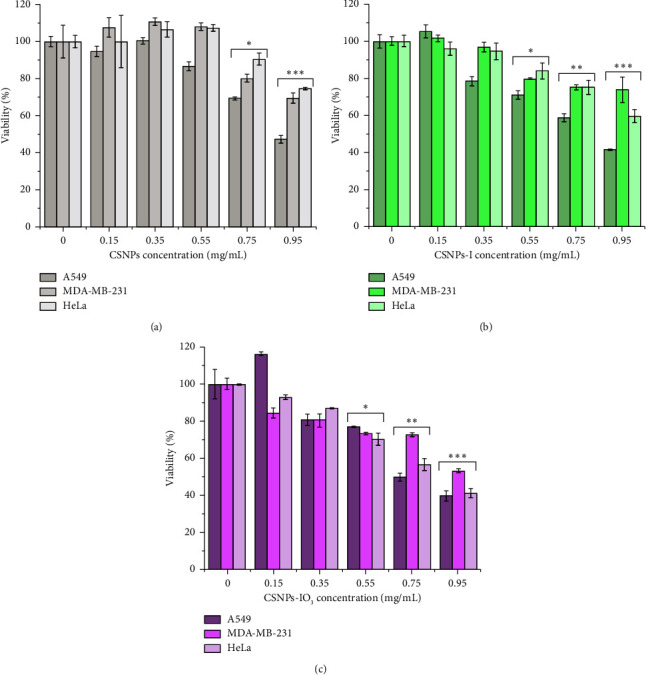
Comparison of cytotoxic effect between tumoral cell lines for each chitosan nanoparticle, CSNPs (a), CSNPs-I (b), and CSNPs-IO_3_ (c). Data were analyzed using two-way ANOVA followed by Dunnett's multiple comparison test. Asterisks denote comparison made to control. ^∗^*p* < 0.05, ^∗∗^*p* < 0.01, ^∗∗∗^*p* < 0.001.

Figure 8 shows the cytotoxic effects of three cell lines for each chitosan nanoparticle (CSNPs, CSNPs-I, and CSNPs-IO_3_). CSNPs at low concentrations (0.15, 0.35, and 0.55 mg/mL) did not have a significant cytotoxic effect in the three cell lines. Still, at high concentrations (0.75 and 0.95 mg/mL), there was a significant cytotoxic effect (^∗^*p* < 0.05, ^∗∗^*p* < 0.01) between cell lines (Figure 7(a)). The cytotoxic effect of CSNPs-I and CSNPs-IO_3_ was significant (^∗^*p* < 0.05,^∗∗^*p* < 0.01,^∗∗∗^*p* < 0.001) in all cancer cell types at concentrations of 0.55, 0.75, and 0.95 mg/mL, see Figures 7(b) and 7(c).

## 5. Conclusions

The NPs synthesized did not present differences in morphology; the three samples showed a hemispherical form with sizes around 30–70 nm. One of the principal differences was observed in thermogravimetric and differential scanning calorimetry analysis, where CSNPs-IO_3_ nanoparticles were less stable with respect to CSNPs and CSNPs-I and with crystallinity index 5% smaller. Finally, the cytotoxic effect on the three cell lines studied (lung cancer A549, cervical cancer HeLa, and breast cancer MDA-MB-231) was more remarkable in CSNPs-IO_3_ nanoparticles, using concentrations at 0.55, 0.75, and 0.95 mg/mL.

## Figures and Tables

**Table 1:** Crystallinity of chitosan nanoparticles and chitosan-iodine nanoparticles (X-ray analysis).

Sample	2*θ* (°)	*d* (A)	*D*[100] (Å)	IC (%)
CS	10.3, 19.9	8.5, 4.4	2.19	69.9
CSNPs	12.5, 23.0	7.0, 3.8	2.36	49.3
CSNPs-I	12.4, 22.3	7.1, 3.9	1.71	48.4
CSNPs − IO_3_	11.0, 24.3	8.0, 3.6	2.08	43.85

**Table 2:** Iodine ion concentration in the chitosan nanoparticles-iodine (*µ*g I^−^ or IO_3_^−^/mg CSNPs).

-		Chitosan nanoparticles–iodine (mg/mL)			
Iodine ion	0.15	0.35	0.55	0.75	0.95
I^−^	2.47	5.76		9.06	12.35
IO_3_^−^	2.38	5.50		8.72	11.89

## Data Availability

The data that support the findings of this study are available on request from the corresponding author.
